# Penile Abscess Complicating Chronic Penile Calciphylaxis in a Heart Transplant Recipient and End-Stage Renal Disease Patient

**DOI:** 10.7759/cureus.85607

**Published:** 2025-06-09

**Authors:** Bharath P Bhushan, Sharath Rajagopalan, Vikash Kumar, David Shi, Eric Huang

**Affiliations:** 1 Internal Medicine, West Virginia University, Morgantown, USA

**Keywords:** end-stage kidney disease, heart transplant patient, partial penectomy, penile calciphylaxis, penile necrosis

## Abstract

Calciphylaxis, also known as calcific uremic arteriolopathy, is a rare but severe and life-threatening condition that is characterized by cutaneous arteriolar calcification and subsequent tissue necrosis. Calciphylaxis is more commonly seen in patients with end-stage renal disease (ESRD) and has a one-year mortality of greater than 50%. Penile calciphylaxis is extremely rare and carries a high mortality risk. Oftentimes, diagnosis and treatment are challenging. We present a case of a 71-year-old heart transplant recipient and end-stage renal disease patient with a history of chronic penile calciphylaxis who developed penile abscesses, highlighting the challenges of managing this complicated condition.

## Introduction

Penile calciphylaxis is a rare and debilitating condition characterized by calcification and necrosis of subcutaneous and dermal tissues. It is often associated with patients who have end-stage renal disease (ESRD) and are hemodialysis (HD)-dependent, although even in this population, it only has an estimated incidence rate between 1% and 4% [1]. Calciphylaxis in general can lead to significant morbidity and mortality, including chronic pain, ulceration, infection, and recurrent hospitalizations. We present a case of a 71-year-old heart transplant recipient with ESRD and penile calciphylaxis who developed a penile abscess complicated by repeated infections. The patient had multiple hospitalizations and ultimately succumbed to his illness. In elucidating the challenges in diagnosing, treating, and managing this patient’s condition, we hope to help future patient diagnosis and management, which will ultimately decrease morbidity and mortality.

## Case presentation

A 71-year-old male patient with a history of orthotopic heart transplant eight months prior, ESRD on hemodialysis, hypertension, hyperlipidemia, and diabetes mellitus presented with increasing pain and foul-smelling discharge from a pre-existing penile wound.

Prior to this presentation, the patient had undergone an orthotopic heart transplant in February 2024 during a lengthy hospitalization initiated for treatment of congestive heart failure. His hospital course included Impella heart pump placement prior to transplant surgery and would require extracorporeal membrane oxygenation (ECMO) initiation after surgery. Immunosuppressive regimen initially included basiliximab induction, mycophenolate mofetil 1,000 mg BID, tacrolimus maintained at goal level 10-12 ng/mL, and prednisone taper. The postoperative course was complicated by necrotizing pneumonia and right lower lobe abscess, during which time mycophenolate mofetil was held for several weeks. Maintenance immunosuppressive regimen would consist of mycophenolate mofetil 500 mg BID and tacrolimus at goal level 8-10 ng/mL. Preoperatively, the patient had a diagnosis of stage 4 chronic kidney disease, and after heart transplantation surgery, he was found to be anuric and was started on continuous renal replacement therapy that was transitioned to intermittent and then scheduled hemodialysis (HD) after lack of renal recovery was apparent. With three-hour HD sessions thrice weekly, the patient was able to achieve adequate dialysis clearance (evidenced by Kt/V 1.2-1.4). Approximately four months after heart transplantation and initiation of hemodialysis, the patient was noted to have the first symptoms that would lead to calciphylaxis diagnosis, with dark discoloration and tenderness noted around the meatus of the glans penis. Sodium thiosulfate 25 mg was started with hemodialysis sessions (thrice per week) and was continued for five months before a change in goals of care. In terms of hyperphosphatemia management, the patient was mostly maintained on sevelamer carbonate 800 mg TID. He was prescribed calcium acetate for a month prior to his presentation with signs of calciphylaxis but appears to have been inconsistently taking this medication due to associated nausea before it was again replaced by sevelamer carbonate. He had also been on vitamin D3 1,000 units daily, which was stopped once calciphylaxis was suspected. The patient never received any calcimimetic agents or vitamin D analogs. The highest intact parathyroid hormone level measured in this patient was 128.1 pg/mL near the time of calciphylaxis diagnosis. It was found to be within a controlled range by his presentation with findings concerning for penile abscess.

**Figure 1:**
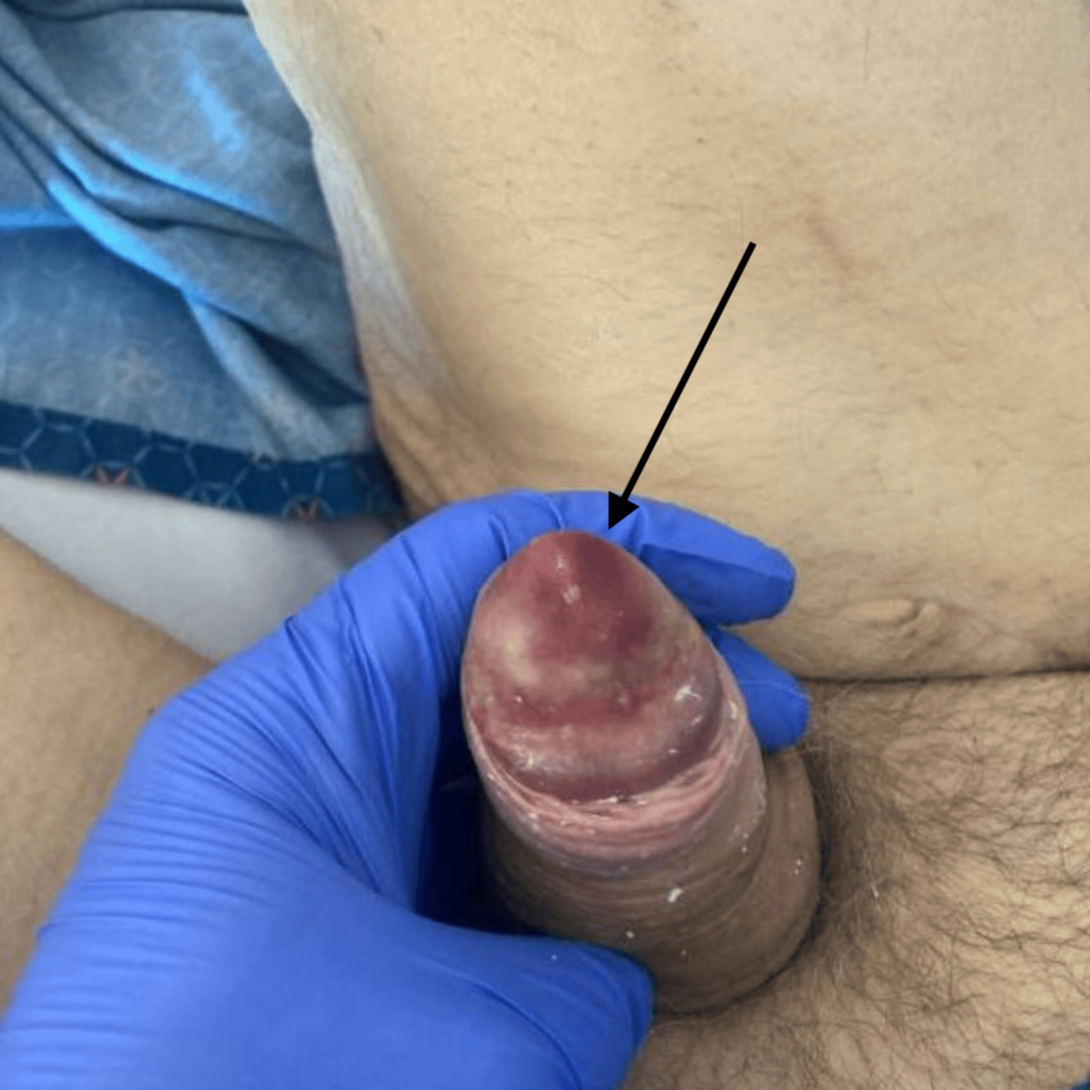
Penile calciphylaxis in a 71-year-old male patient with end-stage renal disease The image shows areas of necrosis (arrow) consistent with calcific uremic arteriolopathy.

**Figure 2:**
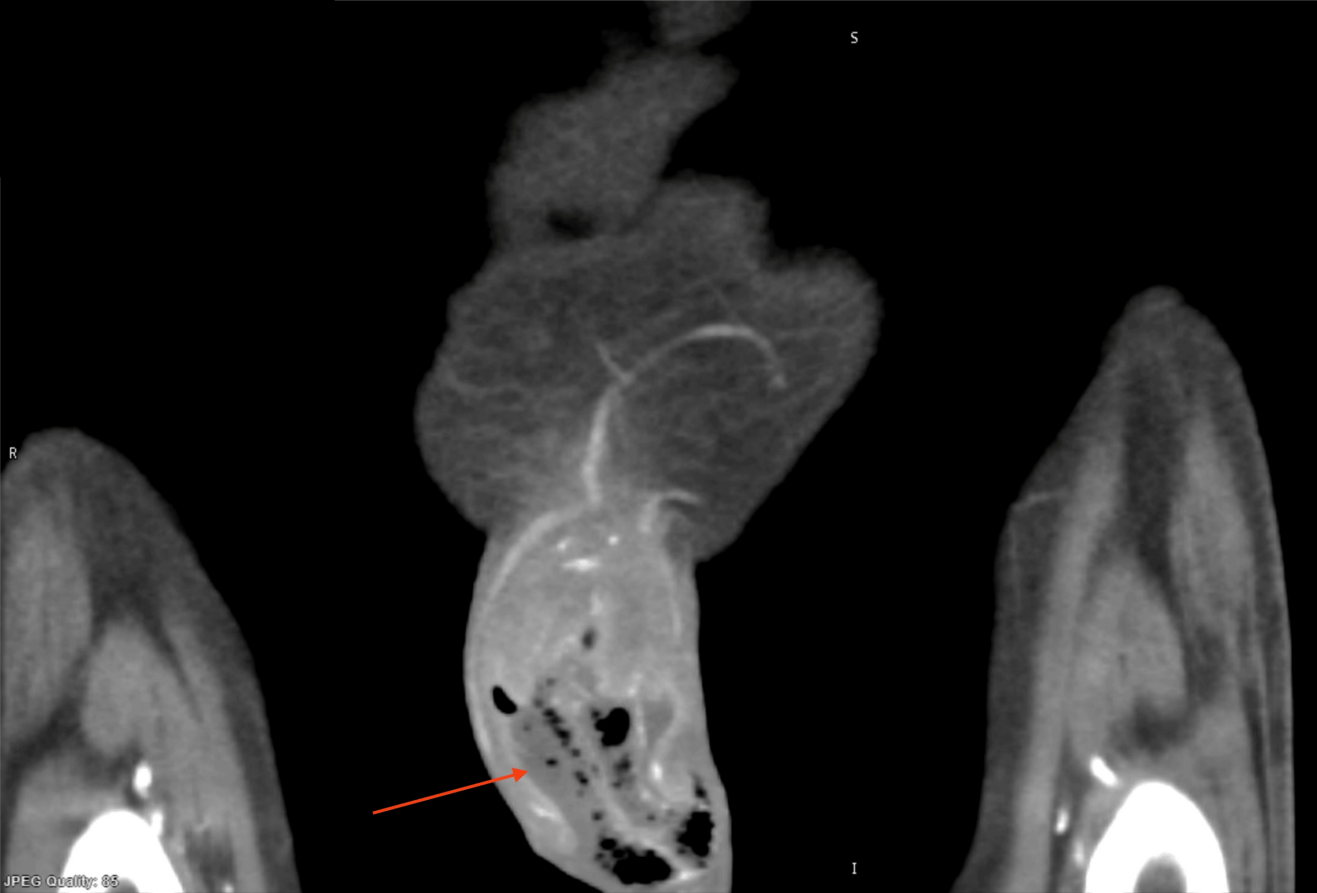
Coronal CT image of the abdomen and pelvis demonstrating a fluid and air collection (arrow) within the distal corpus cavernosum of the penis, with associated penile edema, findings consistent with extensive soft tissue necrosis and abscess formation CT: computed tomography

In the current presentation for progression of penile symptoms, the patient was afebrile and hemodynamically stable but disoriented. Physical examination revealed a necrotic, erythematous, and edematous penile lesion with significant pain and crepitus (Figure 1). Laboratory investigations (Table 1) included elevated white blood cell count, elevated inflammatory markers, and mild hypercalcemia. Computed tomography (CT) imaging confirmed the presence of extensive soft tissue necrosis and abscess formation in the penis (Figure 2).

**Table 1:** Key laboratory values in a patient with penile calciphylaxis and abscess

Laboratory test	Result	Reference range
White blood cell count	23.6 × 10^3^ cells/uL	4.5-11.0 × 10^3^ cells/uL
C-reactive protein	76.4 mg/L	<10 mg/L
Creatinine	5.05 mg/dL	0.6-1.2 mg/dL
Calcium, corrected	11.9 mg/dL	8.5-10.2 mg/dL
Phosphorus	4.2 mg/dL	2.5-4.5 mg/dL
Parathyroid hormone, intact	36.3 pg/mL	10-65 pg/mL

The patient underwent radical excision and debridement of necrotizing soft tissue infection of penis, partial penectomy, anterior urethroplasty (one stage), cystostomy creation, and suprapubic catheter placement. Surgical pathology from partial penectomy demonstrated abscess formation and extensive calcifications suggestive of calciphylaxis. Postoperatively, he was initiated on intravenous antibiotics with meropenem and linezolid for a 14-day course and then transitioned to levofloxacin for prophylaxis in the setting of a suprapubic catheter.

His condition was further complicated by severe protein-calorie malnutrition, for which nocturnal tube feeds were continued via percutaneous endoscopic gastrostomy tube, and a sacral abscess, which required bedside debridement. In line with the goals of the patient and family, once stabilized, he was discharged to an inpatient physical rehabilitation facility. He was readmitted three weeks later for nausea, vomiting, encephalopathy, and adult failure to thrive, during which time the antibiotic regimen was re-escalated with intravenous vancomycin and cefepime. The patient became more lethargic, likely had an aspiration of tube feeds, and had a further decline in respiratory status, which required a high-flow nasal cannula for oxygen supplementation. Ultimately, after discussion with supportive care consultation, the decision was made by his medical power of attorney to transition him to comfort measures only (CMO). He subsequently succumbed to his illness and passed away.

## Discussion

Calciphylaxis, also known as calcific uremic arteriolopathy, is a relatively rare condition with an annual incidence in the United States estimated to be around 35 cases per 10,000 patients undergoing hemodialysis [2]. It involves cutaneous arteriolar calcification, typically in dermis and adipose-rich tissue, with subsequent ischemia and infarction resulting in painful skin lesions [2]. Penile calciphylaxis is an extremely rare and debilitating condition, and due to its rarity, it is often difficult to diagnose. Of patients with calciphylaxis, only an estimated 6% have penile involvement, with a mortality rate near 64%, with a mean time to death of 2.5 months [3].

Diagnosis is made via characteristic history and physical examination, typically consisting of a painful necrotic penile lesion, supported by imaging, and biopsy of an extragenital lesion if available and diagnosis uncertain. Computed tomography may show calcification of penile arteries, and subcutaneous tissue may show fat-stranding or edema. Ultrasound may show hyperechoic linear foci indicative of calcification, leading to heterogenous echotexture of necrosed penile tissue, and with reduced blood flow evidenced by color Doppler. Penile biopsy is recommended against by several authors due to the risk of non-healing and infection [4,5]. Due in part to the low prevalence of the condition, there are no established standard guidelines for the diagnosis and treatment of penile calciphylaxis.

Management typically involves local wound care, symptom management, and sodium thiosulfate to delay progression, although if this conservative approach is unsuccessful, most patients require partial or total penectomy [6]. The mainstay of management is geared toward the prevention of this disorder and associated metabolic derangements. This includes monitoring dialysis efficacy, preventing hypercalcemia, and avoiding calcium-containing phosphate binders. Furthermore, cinacalcet therapy has also been found to effectively reduce the incidence of calciphylaxis in patients with secondary hyperparathyroidism on hemodialysis. Its mechanism of action is linked to the modulation of parathyroid hormone activity and consequent lowering of serum calcium levels, ultimately reducing the risk of calcium depositions [7]. Although hyperphosphatemia and calcium balance appear to contribute, vascular calcification appears to be primarily due to vascular factors that inhibit and promote it and is less likely to be primarily resulting from an increased calcium-phosphate product [8,9]. Sodium thiosulfate is used as an off-label agent for calciphylaxis; in a case series evaluating the efficacy of sodium thiosulfate, more than 70% of the patients receiving it had improvement or resolution of their skin lesions, with a lower mortality rate reported compared to historical data [10]. The mechanism of sodium thiosulfate is believed to be due to the binding of otherwise poorly soluble calcium ions into the dialyzable compound, calcium thiosulfate, as well as vasodilatory properties that improve local blood flow [11]. Despite the prevalent use of sodium thiosulfate, a recent meta-analysis suggests that it may not actually confer significant skin lesion improvement or survival benefits and thus warrants further prospective studies [12].

In this patient’s case, the pre-existing penile calciphylaxis contributed to the development of the penile abscess, which ultimately led to the extensive tissue necrosis requiring surgical intervention.

## Conclusions

This case highlights the challenges of managing complex infections in immunocompromised patients, particularly those with underlying conditions such as penile calciphylaxis. Early recognition and aggressive management, including surgical debridement and appropriate antibiotic therapy, are crucial for improving outcomes in these patients.
